# Age-Friendly Universities (AFU): Necessary Partners for Age-Friendly Communities

**DOI:** 10.1093/geroni/igab046.375

**Published:** 2021-12-17

**Authors:** Joann Montepare

**Affiliations:** Lasell University, Newton, Massachusetts, United States

## Abstract

The pioneering Age-Friendly University (AFU) initiative, endorsed in 2016 by GSA’s Academy for Gerontology in Higher Education (AGHE), calls for institutions of higher education to respond to shifting demographics and the needs of aging populations through more age-friendly campus programs, practices, and partnerships. The case will be made that AFU institutions can also play vital roles in helping neighboring communities develop, launch, assess, and sustain their age-friendly efforts through research and related endeavors that engage students and faculty. In addition, AFU campus-community partnerships can play a critical role in breaking down age-segregation that fuels ageism, building intergenerational connections, and increasing aging literacy among rising community members - all of which are necessary steps for building age-friendly communities.

